# *Toxoplasma gondii* and Schizophrenia

**DOI:** 10.3201/eid0911.030143

**Published:** 2003-11

**Authors:** E. Fuller Torrey, Robert H. Yolken

**Affiliations:** * Stanley Medical Research Institute, Bethesda, Maryland, USA; † Johns Hopkins University Medical Center, Baltimore, Maryland, USA

## Abstract

Recent epidemiologic studies indicate that infectious agents may contribute to some cases of schizophrenia. In animals, infection with *Toxoplasma gondii* can alter behavior and neurotransmitter function. In humans, acute infection with *T. gondii* can produce psychotic symptoms similar to those displayed by persons with schizophrenia. Since 1953, a total of 19 studies of *T. gondii* antibodies in persons with schizophrenia and other severe psychiatric disorders and in controls have been reported; 18 reported a higher percentage of antibodies in the affected persons; in 11 studies the difference was statistically significant. Two other studies found that exposure to cats in childhood was a risk factor for the development of schizophrenia. Some medications used to treat schizophrenia inhibit the replication of *T. gondii* in cell culture. Establishing the role of *T. gondii* in the etiopathogenesis of schizophrenia might lead to new medications for its prevention and treatment.

Schizophrenia is a pervasive neuropsychiatric disease of uncertain cause that affects approximately 1% of the adult population in the United States and Europe. An increased occurrence of schizophrenia in family members of affected persons suggests that genetic factors play a role in its etiology, and some candidate predisposing genes have been identified. Environmental factors are also important. Epidemiologic studies, for example, have established that winter-spring birth, urban birth, and perinatal and postnatal infection are all risk factors for the disease developing in later life. These studies have rekindled an interest in the role of infectious agents in schizophrenia, a concept first proposed in 1896 (*1*). This review focuses on evidence specifically linking infection with *Toxoplasma gondii* to the etiology of some cases of schizophrenia.

*T. gondii* is an intracellular parasite in the phylum Apicomplexa. Its life cycle can be completed only in cats and other felids, which are the definitive hosts. However*, T. gondii* also infects a wide variety of intermediate hosts, including humans. In many mammals, *T. gondii* is known to be an important cause of abortions and stillbirths and to selectively infect muscle and brain tissue. A variety of neurologic symptoms, including incoordination, tremors, head-shaking, and seizures, have been described in sheep, pigs, cattle, rabbits, and monkeys infected with *T. gondii* (*2*).

Humans may become infected by contact with cat feces or by eating undercooked meat. The importance of these modes of transmission may vary in different populations (*3*). Individual response to *Toxoplasma* infection is determined by immune status, timing of infection, and the genetic composition of the host and the organism (*4*).

*Toxoplasma* organisms have also been shown to impair learning and memory in mice (*5*) and to produce behavioral changes in both mice and rats. Of special interest are studies showing that *Toxoplasma-*infected rats become less neophobic, leading to the diminution of their natural aversion to the odor of cats (*6*). These behavioral changes increase the chances that the rat will be eaten by a cat, thus enabling *Toxoplasma* to complete its life cycle, an example of evolutionarily driven manipulation of host behavior by the parasite.

In humans*, Toxoplasma* is an important cause of abortions and stillbirths after primary infection in pregnant women. The organism can also cross the placenta and infect the fetus. The symptoms of congenital toxoplasmosis include abnormal changes in head size (hydrocephaly or microcephaly), intracranial calcifications, deafness, seizures, cerebral palsy, damage to the retina, and mental retardation. Some sequelae of congenital toxoplasmosis are not apparent at birth and may not become apparent until the second or third decade of life. Hydrocephalus (*7*), increased ventricular size (*8*), and cognitive impairment (*9*) have also been noted in some persons with schizophrenia and other forms of psychosis.

Some cases of acute toxoplasmosis in adults are associated with psychiatric symptoms such as delusions and hallucinations. A review of 114 cases of acquired toxoplasmosis noted that “psychiatric disturbances were very frequent” in 24 of the case-patients (*10*). Case reports describe a 22-year-old woman who exhibited paranoid and bizarre delusions (“she said she had no veins in her arms and legs”), disorganized speech, and flattened affect; a 32-year-old woman who had auditory and visual hallucinations; and a 34-year-old woman who experienced auditory hallucinations and a thought disorder (*11*). Schizophrenia was first diagnosed in all three patients, but later neurologic symptoms developed, which led to the correct diagnosis of *Toxoplasma* encephalitis.

Psychiatric manifestations of *T. gondii* are also prominent in immunocompromised persons with AIDS in whom latent infections have become reactivated. Reviews of such AIDS cases with toxoplasmosis have indicated that altered mental status may occur in as many as 60% of patients and that the symptoms may include delusions, auditory hallucinations, and thought disorders (*12*).

Additional studies have documented that persons with serologic evidence of *Toxoplasma* infection have evidence of psychiatric changes in the absence of a history of clinically apparent *Toxoplasma* infection. Studies in which personality questionnaires have been administered to healthy adults have indicated that serum antibodies to *T. gondii* are associated with alterations in behavior and psychomotor skills (*13*). Seropositivity to *Toxoplasma* has also been associated with “lack of energy or tiredness” in schoolchildren (*14*). In view of these findings, we decided to carry out serologic and other studies and to survey the literature for possible additional links between *Toxoplasma* infection and schizophrenia.

## Serologic Studies of Patients with Schizophrenia

### Studies Done Before 1980

In the course of doing our studies, we discovered that much research had been published in languages other than English and was not listed on searchable databases. Through direct contact with authors and by obtaining references listed on their papers, we identified 13 relevant studies published between 1953 and 1979 (*15*–*27*), as listed on the Table. Some publication bias is likely, since negative studies are less likely to have been submitted or published.

**Table:** Toxoplasmosis antibody studies of psychiatric patients

Y	Author and country		Test used	Patients	Controls	% Patients antibody positive	% Controls antibody positive	p value chi square
Before 1980								
1953	Kozar (15) Poland		Skin test	Psychiatric inpatients, all diagnoses	Healthy persons, ages 18–60	52 (495/961)	25 (170/681)	<0.0001
1956	Vojtechovská et al. (16) Czechoslovakia		Skin test	Inpatients with “psychosis”	General population	59 (68/116)	30 (not specified)	<0.0001
1956	Wende (17) East Germany		Dye test	Inpatients with schizophrenia	Inpatients with neurologic disorders	8 (3/38)	5 (24/520)	0.418
1957	Jirovec et al. (18) Czechoslovakia		Skin test	Inpatients with schizophrenia	Normal population	48 (238/501)	29 (286/970)	<0.0001
1958	Buentello (19) Mexico		Color change in fish	Inpatients with schizophrenia	Normal subjects	69 (29/42)	0 (0/60)	<0.0001
1958	Caglieris (20) Italy		Dye test	Inpatients with schizophrenia	Normal subjects	21 (13/61)	15 (12/81)	0.376
1961	Cook & Derrick (21) Australia		Dye test C.F.	Inpatients with schizophrenia	General population	36 (19/53) ≥1:16 11 (6/53) ≥1:4	24 (182/760) 13 (99/760)	0.053 0.840
1962	Yegerov et al. (22) Russia		Skin test C.F.	Inpatients with schizophrenia	Hospital employees	19 (7/37) 32 (12/37)	4 (1/25) 28 (7/25)	0.124 0.784
1962	Avlavidov (23) Bulgaria		Skin test C.F.	Psychiatric inpatients, not specified	Female surgical patients	26 (5/19) 21 (3/14)	3 (1/35) 9 (3/34)	0.017 0.339
1966	Berengo et al. (24) Italy		Dye test	Inpatients with schizophrenia	General population	14 (76/560)	4% (49/1200)	<0.0001
1966	Roch & Varela (25) Mexico		Dye test	Schizophrenia, hospital status not specified	General population	86% (836/973)	30% (4,411/14,689)	<0.0001
1968	Garrido & Redondo (26) Spain		C.F.	Inpatients with schizophrenia	General population	44 (17/39)	29 (147/500)	0.072
1979	Garcia (27) Cuba		Skin test	Psychiatric inpatients	Normal persons	60 (60/100)	30 (30/100)	<0.0001
Since 1999								
1999	Qiuying et al. (28) China		EIA	Inpatients with schizophrenia	Normal persons from same region for routine physicals	14 (22/152)	10 (41/396)	0.181
2001	Gu et al. (29) China		EIA	First-episode schizophrenia	Normal controls matched for age, sex, birthplace	33 (45/135)	9 (4/43)	0.002
2001	Yolken et al. (30) Germany		EIA: IgG or IgM	First-episode schizophrenia	Normal controls matched for age, sex, SES	42 (16/38)	11 (3/27)	0.007
2002	Boronow et al. (31) United States		EIA	Outpatients with schizophrenia	Normal controls matched for age	12 (28/229)	7 (7/100)	0.147
2003	Leweke et al. (32) Germany		EIA	First-episode schizophrenia, never treated	Normal controls matched for age, sex, SES	36 (13/36)	14 (10/73)	<0.007
2003	Torrey & Yolken (unpub. data) Ireland		EIA	Inpatients with schizophrenia	Hospital employees	60 (31/52)	45 (9/20)	0.299

C.F., complement fixation; EIA, enzyme immunoassay; Ig, immunoglobulin; SES, socioeconomic status.

The 13 studies used a variety of immunologic methods for measuring antibodies, including the Sabin Feldman dye test, skin tests, and complement fixation (CF). One study used a test in which an alkaloid from *T. gondii* caused a tropical fish, *Lebistes reticulatus*, to change color (*19*). Some of the studies compared the relative efficacy of two different tests. Most of the studies defined *Toxoplasma-*positive results as the presence of a skin reaction or antibodies above a certain titer but often without specifying the precise details of the method; thus, comparing the older studies with each other was not possible. Most of these studies also did not specify what diagnostic criteria were used for schizophrenia, but since at least 12 of them used inpatients, the patients likely had a severe psychiatric disorder. Similarly, most of the studies did not specify the origin of their control group other than saying such things as “681 healthy persons working or studying in the city of Gdansk” (*15*).

Despite these limitations, 12 of the 13 studies found that the patient group had a higher percentage of antibodies to *Toxoplasma* than the control group. In eight of the studies, the increase was statistically significant by chi square at the level of p <0.05. In the two largest studies, Kozar (*15*) in Poland reported antibodies in 495 (52%) of 961 psychiatric inpatients compared with 170 (25%) of 681 controls, and Roch and Varela (*25*) in Mexico found antibodies in 836 (86%) of 973 patients with schizophrenia compared with finding antibodies in 30% of the general population.

### Studies Since 1999

We identified no studies that were done between 1979 and 1999. Since that time, six studies have been carried out, including our own (*28*–*32*). All used enzyme immunoassay methods for measuring antibodies to *Toxoplasma.* All of the studies also used modern diagnostic criteria for schizophrenia; three studies included patients with chronic disease, and three included patients who were in the first episode of the disease. All of the studies identified their control groups, and some attempts were made to match them to the patient groups.

The results of these studies are summarized in the Table. In all of the studies, the patients had more antibodies to *Toxoplasma* than the control groups, and in the three studies, carried out in China and Germany, of patients who were having their first-episode of schizophrenia, the differences were statistically significant. One of the first-episode studies, carried out in Cologne by Leweke et al. (*32*), divided the first-episode patients into those who had never received antipsychotic treatment and those who had received some treatment. The antibody levels for the treated group were intermediate between the levels of the never-treated group and those of the control group, suggesting that antipsychotic medication may have decreased the antibody levels. This conclusion is supported by a study that indicated that some antipsychotic medications inhibit the growth of *T. gondii* in cell culture (*33*).

The Leweke et al. study also collected cerebrospinal fluid (CSF) from the first-episode patients. The level of *Toxoplasma* antibody in the CSF of untreated patients was significantly higher than the normal controls (p<0.0001) (*32*). Treated first-episode patients had CSF antibody levels intermediate between those of the untreated patients and the controls, just as was found for the sera.

In addition to these studies on adults with schizophrenia, a study was also conducted by analyzing on serum samples from pregnant women, obtained shortly before delivery, who gave birth to children in whom schizophrenia or other psychoses developed. Preliminary analysis indicates an increased rate of immunoglobulin (Ig) M (but not IgG) class antibodies to *Toxoplasma gondii* in mothers with infants in whom schizophrenia developed later, suggesting that the mothers were experiencing an active infection or that they had persistent IgM antibodies, as described in other studies. Increased levels of IgM antibodies were not found to other perinatal pathogens such as rubella virus or cytomegalovirus (*34*).

## Discussion

Multiple studies have demonstrated that the brains of persons with schizophrenia show structural and functional changes and that these exist even in patients who have never been treated with antipsychotic medications (*35*). Thus, schizophrenia, like multiple sclerosis and Parkinson disease, is a chronic disease of the central nervous system; as with other such diseases, infectious agents should be considered as possible etiologic agents, perhaps in persons who also have an increased genetic susceptibility.

*T. gondii* is of special interest because of its known affinity for brain tissue and its capacity for long-term infection starting in early life. The effect of *Toxoplasma* infection on any given person may differ, depending on such factors as individual genetic predisposition, the state of the immune system, the dose, the virulence of the infecting strain, the timing (e.g., infections in the first trimester of pregnancy differ from those in the third trimester; prenatal and postnatal infections differ; etc.), and the part of the brain affected.

If *Toxoplasma* is involved in the etiology of schizophrenia, however, its synergy with genes may determine the person’s brain development, immune response to infections, and response to other infectious agents. The fact that *T. gondii* has been shown to activate retroviruses in animal model systems may be relevant (*36*). This property is consistent with the recent finding that many persons with schizophrenia exhibit increased retroviral activation within their central nervous systems (*37*).

Numerous studies indicate that, although the symptoms of schizophrenia generally do not manifest until late adolescence or early adulthood, the disease process has its origins in earlier stages of brain development. The ability of *Toxoplasma* organisms to infect the perinatal brain is thus consistent with this aspect of schizophrenia pathogenesis. However, prospective studies also support a possible role of postnatal infections in some cases of schizophrenia (*38*). The potential effects of the transmission of *Toxoplasma* in early childhood or later in life should thus be considered.

Epidemiologically, two studies have reported that adults who have schizophrenia or bipolar disorder had a greater exposure to cats in childhood. In one study, 84 (51%) of the 165 affected versus 65 (38%) of the 165 matched controls had owned a house cat in childhood (p = 0.02) (*39*). In the other study, 136 (52%) of the 262 affected versus 219 (42%) of the 522 matched controls owned a cat between birth and age 13 (odds ratio 1.53; p<0.007) (*40*). Whether any geographic association exists between the prevalence of toxoplasmosis and the prevalence of schizophrenia is unknown. France, which has a high prevalence of *Toxoplasma-*infected persons, was reported to have first-admission rates for schizophrenia approximately 50% higher than those in England (*41*). Ireland also has a high rate of *Toxoplasma-*infected persons in rural areas (*42*), confirmed by the high rate of infection in hospital personnel in our own study. The area of our study in Ireland has also been reported to have a high prevalence of schizophrenia (*43*).

Neuropathologically, studies of *T. gondii* in cell culture have shown that glial cells, especially astrocytes, are selectively affected (*44*,*45*). Postmortem studies of schizophrenic brains have also reported many glial abnormalities (*46*), including decreased numbers of astrocytes (*47*). Similarly, animal studies of *Toxoplasma* infections have demonstrated that this organism affects levels of dopamine, norepinephrine, and other neurotransmitters, which are well known to be affected in persons with schizophrenia.

Few data exist concerning the clinical correlates of *Toxoplasma* infection in persons with schizophrenia. A recent study found that persons with schizophrenia who have serologic evidence of *Toxoplasma* infection have increased levels of cognitive impairment compared to age-matched *Toxoplasma*-seronegative patients with similar degrees of psychotic symptoms (*31*). Additional studies are needed on the possible associations between *Toxoplasma* infections and the symptoms or clinical course of schizophrenia and other psychiatric diseases.

One limitation of studies of *Toxoplasma* infection and schizophrenia is that one cannot conclusively rule out disease-related differential exposure to the organism. Thus, hospitalized patients may be fed undercooked meat, thereby increasing their seropositivity. Alternatively, the authors of one of the studies speculated that the increased patient seropositivity might have been because the patients worked in the hospital gardens, which were also frequented by cats (*21*). The possible effects of hospitalization, altered behavior, or other artifactual factors on seropositivity can be minimized by the analysis of persons with the recent onset of symptoms, as three studies described above have done.

Studies are ongoing in attempts to better define the relationship of *Toxoplasma* infection to schizophrenia. An initial study of the orbital frontal cortex of 14 persons with schizophrenia (*48*), in which primers to *T. gondii* were used*,* did not detect sequences. Studies should also include organisms such as *Neospora caninum* and *Hammondi hammondi,* which are closely related to *T. gondii* and which cross-react serologically (*49*); *N. caninum* has been detected in human specimens in our laboratory and by others (*50*). The use of organism-specific antigens generated from molecular cloning and the use of stage-specific antibodies should help elucidate both the specificity and the timing of the infection.

Finally, clinical trials are under way of antimicrobial drugs with anti-*Toxoplasma* activity, such as trimethoprim-sulfamethoxazole and azithromycin, as adjunctive treatment for persons with schizophrenia in double-blind trials. These studies may lead to new methods for the treatment of schizophrenia and other psychiatric disorders that may be associated with *Toxoplasma* and related organisms.
